# Burden and unmet need for specialist care in poorly controlled and severe childhood asthma in a Danish nationwide cohort

**DOI:** 10.1186/s12931-023-02482-7

**Published:** 2023-06-27

**Authors:** Kjell Erik Julius Håkansson, Silvia Cabrera Guerrero, Vibeke Backer, Charlotte Suppli Ulrik, Deepa Rastogi

**Affiliations:** 1 Department of Respiratory Medicine, Copenhagen University Hospital-Hvidovre, Kettegård Allé 30, 2650 Hvidovre, Denmark; 2 Division of Pulmonary and Sleep Medicine, Children’s National Health System, 111 Michigan Ave NW, Washington, DC 20010 USA; 3 Department of Otorhinolaryngology, Copenhagen University Hospital-Rigshospitalet, Blegdamsvej 9, 2100 Copenhagen, Denmark; 4 Institute of Clinical Medicine, University of Copenhagen, Blegdamsvej 3B, 2200 Copenhagen, Denmark; 5 Pediatrics, Genomics and Precision Medicine, George Washington University School of Medicine and Health Sciences, 2300 I St NW, Washington, DC 20052 USA

**Keywords:** Paediatric asthma, Population cohort, Exacerbations

## Abstract

**Background:**

Asthma is a common disease in childhood and adolescence with lifelong consequences particularly among those at risk of severe disease, poor control and/or frequent exacerbations. Specialist care is recommended for at-risk children and adolescents, yet access to specialist management in free-to-access healthcare settings remains poorly understood.

**Methods:**

A Danish nationwide cohort of children and adolescents aged 2–17 years with persistent asthma, defined as repeated redemption of inhaled corticosteroids (ICS) during 2015, were followed for two years, to identify at-risk children and adolescents comprising those with severe asthma (classified according to GINA 2020 guidelines), poor control (defined as use of 400/600 (ages 2–11/12 +) annual doses of short-acting bronchodilators), or frequent exacerbations (defined as use of oral steroids or hospitalization), and access to specialist care. The population is chosen due to detailed medical records in the setting of universal health care.

**Results:**

The cohort comprised of 29,851 children and adolescents (59% boys), with a median age of 9 years. While 17% of children were on high dose ICS, 22% were on daily ICS below GINA low dose cut-off. Prevalence of severe asthma (3.0–6.5%) was lower than poor asthma control (6.4–25%); both declined from childhood to adolescence. Exacerbations occurred in 7.1–9.0% of children, with median number of exacerbations being 1 (IQR 1–1). Despite being classified as having mild-to-moderate asthma, 15% had poor asthma control and 3.8% experienced exacerbation(s), respectively. While 61% of children with severe asthma and 58% with exacerbation-prone disease were in specialist care, only 24% with uncontrolled disease were receiving specialist care. Of children and adolescents using high-dose ICS, 71% were managed in primary care, while the use of additional controllers was more common in specialist care.

**Conclusions:**

Throughout childhood and adolescence, there was a high prevalence of severe asthma and poor control, although their prevalence declined with age. We demonstrate a large unmet need for specialist care among children with at-risk asthma, particularly among those with poorly controlled asthma, even in a system with free-to-access, tax-funded healthcare.

**Supplementary Information:**

The online version contains supplementary material available at 10.1186/s12931-023-02482-7.

## Introduction

Asthma is the most prevalent chronic lung disease among children and adolescents worldwide, with an estimated prevalence of 11.7% in Denmark [1]. Poorly controlled and severe asthma in children has a substantial impact on quality of life due to missed school days, impaired academic performance, limited physical activity, and participation in school activities [2], and impact on parental attendance at work [3]. Specialist care for children with poorly controlled and severe asthma can improve long-term outcomes [4, 5], but there is limited understanding of impact of access and effectiveness of subspecialty care on disease severity and its poor control in settings with universal health care.

The prevalence of severe asthma and poor disease control varies widely across geographic locations, ages, and ethnicities, and by definitions outlined by different organizations, many of which are based on medication level needed to achieve control. Although most children achieve symptom control with low to medium doses of inhaled corticosteroids (ICS) [3], children with severe asthma or difficult-to-control asthma require higher doses of ICS or alternative therapies to maintain symptom control [6]. Prevalence of severe disease in adults with asthma is estimated at 5–10% [7] by the European Respiratory Society and the American Thoracic Society, whereas the prevalence of severe disease in children is less certain [7, 8]. A retrospective multicentre cohort study identified a prevalence of severe paediatric asthma ranging from 1.6 to 18.3% across six European electronic healthcare databases [9], whereas poorly controlled asthma has been reported in up to 41% children aged 5–16 years in UK primary care [10]. In addition, the episodic nature of childhood asthma contributes to a set of children who have frequent or severe exacerbations but do not fit the criteria of severe asthma or poor disease control as per the Global Initiative for Asthma (GINA) guidelines. Exacerbation-prone paediatric asthma that is not severe or poorly controlled asthma is not well understood [11, 12].

Given the impact of severe, as well as poorly controlled, childhood asthma on quality of life, guidelines recommend that its management should include specialist expertise to ensure achievement of disease control, with the goal to prevent long-term, irreversible changes in lung function [3]. Despite the beneficial effects of specialist care for both uncontrolled and severe childhood asthma [4, 5], and access to novel biologic therapies being exclusive to specialised centres [13], little is known about the extent of specialist care in these vulnerable populations, especially in a setting with tax-funded, free to access healthcare.

In the present study, using nationwide Danish databases, we address these gaps in knowledge and aim to elucidate the prevalence of possible severe asthma, poorly controlled asthma and exacerbation-prone disease among children and adolescents aged 2–17 years, as well as access to specialist care in a tax-funded, free to access healthcare system.

## Methods

### Data sources

The Uncontrolled and Possible Severe Asthma in Denmark (REASSESS) Youth cohort is a nationwide Danish cohort identified by utilizing universal linkage between multiple data sources. Using the Central Person Registry numbers assigned at birth or immigration, individual-level data can be cross-referenced across most aspects of public resource utilization such as healthcare, welfare, and social security [14]. Data was provided by Statistics Denmark, the Danish National Prescription Database, the national quality of care register RKKP-DrAstma and the National Patient Registry.

**Fig. 1:**
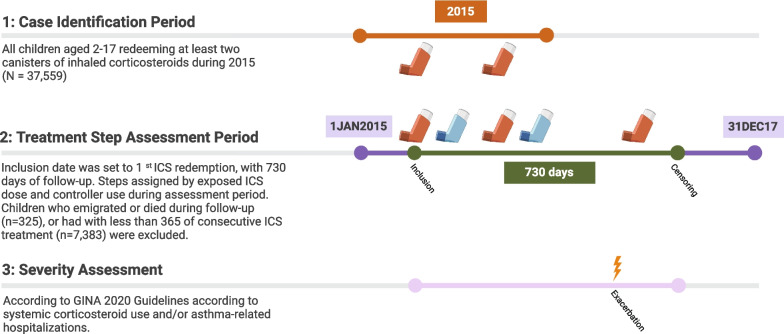
Case identification, treatment intensity and severity assessment in a Danish nationwide cohort of children aged 2–17 years with actively treated asthma

Danish children and adolescents aged 2–17 years who redeemed two or more canisters of inhaled corticosteroids (ICS) in a calendar year during 2015 were considered to have actively treated asthma and were included in the present study. The cohort was divided into three age categories (2–5 years, 6–11 years, and 12 + years) according to the GINA 2020 guidelines [15]. Age was defined as age on the index date, and children were followed from their index date (date of 1st ICS redemption) prospectively for 730 days. Children who emigrated, died or had less than 365 days of continuous ICS treatment were excluded (Fig. 1).

### Asthma treatment, severity, and disease control measures

GINA 2020 Treatment Steps were used to characterise asthma for each age group [15]. ICS treatment was calculated as the average daily dose (calculated as beclomethasone standard-particle dose equivalents) during the observation period as previously described [16] and stratified as Below Low, Low, Moderate, or High dose according to the age-appropriate GINA 2020 dosing chart [15]. ATC-codes used for identifying ICS use and any additional controllers, as well as ICS strength equivalence calculations can be found in the Additional file 1: Tables S1 and S2.

Exacerbation-prone asthma was defined as either two moderate or one severe/near-fatal exacerbation(s). Severe asthma was defined as Exacerbation-prone asthma treated with GINA 2020 Step 3 or 4 for individuals aged 2–11 years. For individuals aged 12 + years, criteria included exacerbation-prone asthma treated with GINA Step 4 treatment, or GINA Step 5 treatment regardless of exacerbations. Mild-to-moderate asthma was defined as any treatment intensity not fulfilling above criteria for severe disease.

Disease control was defined based on the total number of redeemed doses of short-acting β-agonist (SABA) use during the follow-up period, and is presented as annualised redeemed doses. Excessive SABA use was defined as twice the acceptable control criteria for children aged 11 and below (two daily puffs at least two days a week, totalling 400 annual doses) or 600 (ages 12 +)) annual redeemed doses of SABA [15, 17].

With regard to exacerbations, moderate exacerbations were defined as redemption of at least 37.5 mg of prednisolone for 5 days, severe exacerbations as hospitalisation (International Classification of Diseases (ICD)-10 codes DJ45, DJ46, DJ96, DJ960 or DJ969) and near fatal exacerbations as hospitalisation (ICD-10 codes as above) to an intensive care unit with or without ventilation. Re-exacerbations within 14 days were considered to be treatment failures and did not count as renewed exacerbation burden. Exacerbations were ranked by severity, e.g., a moderate exacerbation followed by a hospital admission within 14 days would be registered as a single severe exacerbation event.

Specialist management of asthma was defined as an active outpatient A-level ICD-10 code (DJ45, DJ46) during the observation period.

### Statistical analyses

The primary outcome of interest was quantifying the prevalence of severe asthma, poorly controlled asthma, and exacerbation-prone asthma in the REASSESS: Youth cohort. The cohort was characterized using demographic statistics presented as median (interquartile range, IQR). Wilcoxon rank-sum test or χ^2^-test were used depending on continuous or categorical data for groupwise comparisons. R 4.1.0 (The R Foundation, AU) was used for statistical analyses; Ggplot2 and Biorender were used for creating figures.

## Results

The study cohort of 29,851 children and adolescents receiving active asthma treatment and followed for two years was comprised of 59% boys and 41% girls. Among these, 17%, 30% and 3.1% of children and adolescents aged 2–5, 6–11 and 12+ years were on high dose ICS, respectively. Long-acting beta_2_-agonist (LABA) use increased with age, reaching 45% among adolescents aged 12+ years, while leukotriene receptor antagonist (LTRA) use was relatively constant across all ages at 22% (Table 1). When stratified according to the GINA 2020 Treatment Steps, children aged 2–5 demonstrated a tapering spread between Steps 2 through 4, whereas Steps 3 and 4 were the most common steps in children aged 6–11 years at 34% and 37%, respectively. For adolescents, a trend towards the lower Steps were seen, with only 11 and 2.1% of patients belonging to Steps 4 and 5, respectively (Table 1).

**Table 1:** Demographics, comorbidities, and treatment of 29,851 children aged 2–17 years with actively treated asthma from a nationwide cohort

	Overall (N = 29,851)	Ages 2–5 yrs (N = 10,105)^a^	Ages 6–11 yrs (N = 9895)^a^	Ages 12–17 yrs (N = 9851)^a^
Age	9.0 (4.0, 13.0)	3.0 (2.0, 4.0)	9.0 (7.0, 10.0)	15.0 (13.0, 16.0)
Male	17,646 (59%)	6067 (60%)	6362 (64%)	5217 (53%)
Specialist asthma care	5864 (20%)	1536 (15%)	2295 (23%)	2033 (21%)
GINA 2020 step				
Step 1	N/A	N/A	944 (9.5%)	2921 (30%)
Step 2	N/A	7895 (52%)	1887 (19%)	2881 (29%)
Step 3	N/A	4736 (31%)	3371 (34%)	2797 (28%)
Step 4	N/A	2497 (17%)	3693 (37%)	1042 (11%)
Step 5	N/A	N/A	N/A	210 (2.1%)
ICS dose				
Below low	7111 (22%)	3883 (26%)	1107 (11%)	2121 (27%)
Low	9541 (32%)	2601 (26%)	2068 (21%)	4872 (49%)
Moderate	8905 (30%)	3368 (33%)	3787 (38%)	1750 (18%)
High	4958 (17%)	1717 (17%)	2933 (30%)	308 (3.1%)
Average daily exposed dose (mcg beclomethasone)	247 (134, 401)	201 (107, 329)	267 (164, 406)	280 (166, 468)
Add-on therapies				
Long-acting beta_2_-agonists	6894 (23%)	344 (3.4%)	2125 (21%)	4425 (45%)
Long-acting antimuscarinics	70 (0.2%)	13 (0.1%)	7 (< 0.1%)	50 (0.5%)
Leukotriene receptor antagonists	6439 (22%)	2248 (22%)	2166 (22%)	2025 (21%)

*GINA* Global Initiative for Asthma, *ICS* inhaled corticosteroids

^a^Statistics presented: n (%); median (IQR)

### Prevalence of severe asthma

Severe asthma was seen in 4.9% and 6.5% of children aged 2–5, 6–11 and 3.0% of adolescents aged 12 + years, respectively. While prevalence of severe asthma declined in adolescence, it should be noted that when considered without the GINA Step-classification dependent on exposed ICS doses, 4.5% of adolescents aged 12 + years fulfilled the severe asthma exacerbation criteria (“exacerbation-prone asthma”) and 7.1% experienced at least one exacerbation (Table 2).

**Table 2:** Disease control, exacerbation burden and prevalence of severe asthma for 29,851 children aged 2–17 years with actively treated asthma from a nationwide cohort

		Overall (N = 29,851)	Ages 2–5 years (N = 10,105)^a^	Ages 6–11 years (N = 9895)^a^	Ages 12–17 years (N = 9851)^a^
Asthma control					
Median annual SABA use		200 (100, 360)	300 (100, 500)	200 (100, 300)	100 (30, 250)
High annual SABA use^b^		4750 (16%)	2566 (25%)	1558 (16%)	626 (6.4%)
of which in specialist care		1320 (28%)	603 (23%)	515 (33%)	202 (32%)
Exacerbations^c^					
Any exacerbation(s)		2353 (7.9%)	757 (7.5%)	895 (9.0%)	701 (7.1%)
of which in specialist care		1354 (58%)	437 (58%)	530 (59%)	387 (55%)
Number of exacerbations		1 (1, 1)	1 (1, 1)	1 (1, 2)	1 (1, 2)
Moderate exacerbation(s)		616 (2.1%)	74 (0.7%)	182 (1.8%)	360 (3.7%)
Number of exacerbations		1 (1, 1)	1 (1, 1)	1 (1, 1)	1 (1, 1)
Severe exacerbation(s)		1807 (6.1%)	675 (6.7%)	739 (7.5%)	393 (4.0%)
Number of exacerbations		1 (1, 1)	1 (1, 1)	1 (1, 1)	1 (1, 1)
Near fatal exacerbation(s)		64 (0.2%)	30 (0.3%)	18 (0.2%)	16 (0.2%)
Number of exacerbations		1 (1, 1)	1 (1, 1)	1 (1, 1)	1 (1, 1)
Exacerbation-prone asthma^d^		1910 (6.4%)	699 (6.9%)	767 (7.8%)	444 (4.5%)
Possible severe asthma^e^		1430 (4.8%)	496 (4.9%)	642 (6.5%)	292 (3.0%)
of which in specialist care		868 (61%)	316 (64%)	418 (65%)	134 (46%)

*GINA* Global Initiative for Asthma, *SABA* Short-acting bronchodilator

^a^Statistics presented: n (%); median (IQR)

^b^Defined as > 400 annual doses of SABA for ages 0–11, > 600 for ages 12 and above

^c^Moderate exacerbations defined as redemption of oral prednisolone at 37.5 mg for at least 5 days, severe as hospitalization with an asthma diagnosis and near-fatal as admission to an intensive care unit with an asthma diagnosis. Number of exacerbations denote the median number of exacerbations per exacerbating child during the follow-up period

^d^Defined as two moderate or one severe exacerbation(s) during the follow-up period

^e^Defined as exacerbation-prone asthma with GINA Step 3 + 4 treatment for ages 0–11, exacerbation-prone GINA Step 4 or GINA Step 5 regardless of exacerbations for ages 12 and above

### Prevalence of poor disease control

Median SABA use, based on annualised redeemed puffs of SABA, ranged from 300 (100, 500) to 100 (30, 250) in children aged 2–5 and adolescents, respectively. Poor disease control, as assessed by excessive SABA use, was present in 25%, 16% and 6.4% of children aged 2–5, 6–11 years and adolescents aged 12 + years, respectively (Table 2).

### Prevalence of exacerbations

The prevalence of children and adolescents experiencing exacerbation(s) was 7.9% overall, with children aged 6–11 showing the highest prevalence at 9.0%. Most exacerbations were classified as severe, irrespective of age group, and 64 (0.2%) children experienced a near-fatal exacerbation. Of note, a shift from a predominance of severe, hospitalization-requiring exacerbations in younger children, towards an equal distribution between moderate and severe exacerbations in adolescents was seen (Table 2).

### Overlap between clinical phenotypes

**Fig. 2:**
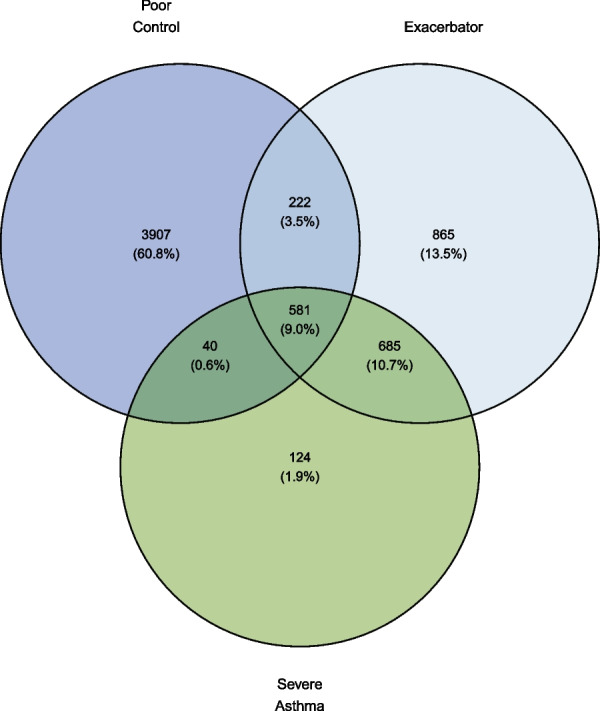
Venn diagram showing the overlap between clinical phenotypes of uncontrolled asthma, asthma exacerbations and severe asthma in 6424 children aged 2–17 years with actively treated asthma. Note that the diagram only includes the subpopulation of children fulfilling at least one of the criteria above. Uncontrolled asthma was defined as > 400 annual doses of SABA for ages 0–11, > 600 for ages 12 and above, asthma exacerbations was defined as redemption of either 37.5 mg prednisolone for 5 days or hospital admission with asthma. Severe asthma was defined as GINA Step 3 + 4 treated asthma with two moderate or one severe/near-fatal exacerbation(s) for ages 0–11, GINA Step 4 treated asthma with two moderate or one severe/near-fatal exacerbation(s) or GINA Step 5 regardless of exacerbations for ages 12 and above

Cumulatively, 21.5% of children and adolescents in the cohort were classified to have at-risk asthma, defined as either severe asthma, uncontrolled asthma, or exacerbation-prone asthma. It is notable that distinct populations of clinical phenotypes such as exacerbation-prone (3.8%) and uncontrolled asthma (15%), are present in children who do not fall within the definition of severe asthma (Fig. 2).

### Effect of age and sex on treatment intensity and disease control

When stratified by sex and age, no significant differences between sexes were observed for assigned ICS dose categories, yet for adolescents a statistically significant skew towards slightly higher GINA scoring was found, driven by increased use of LABA and LAMA among adolescent girls. Statistically significant, but clinically insignificant, differences in ICS exposure measured in micrograms were found for children aged 2–11 years (Additional file 1: Table S3). An age-driven shift from male-dominant to female-dominant prevalence of children experiencing exacerbation(s) during the follow-up period was observed. A similar sex and age-dependent shift in the prevalence of severe asthma was seen (Additional file 1: Table S4).

### At-risk asthma and access to specialist care

**Fig. 3:**
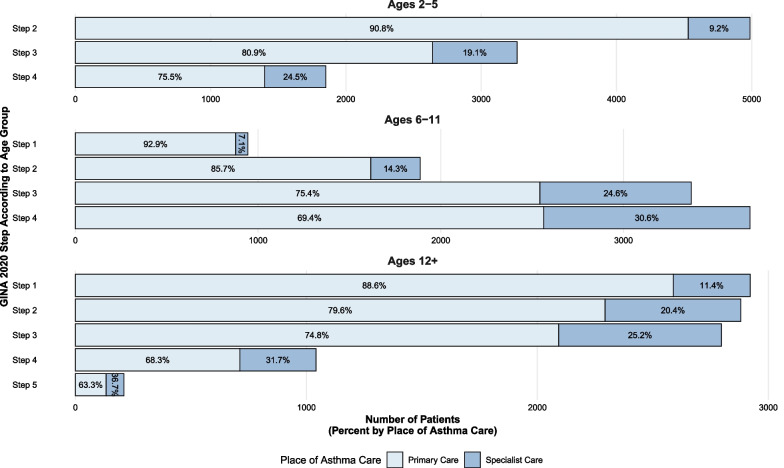
Distribution of GINA 2020 treatment steps in a nationwide cohort of 29,851 children aged 2–17 years with actively treated asthma, stratified by age and place of asthma management

Overall, 20% of children and adolescents were managed by a paediatrician during their follow-up period, with the prevalence increasing with GINA 2020 Steps (Fig. 3). When stratified by sex and age, no significant differences in the prevalence of specialist care were found (Additional file 2: Fig. S1). Among the at-risk children and adolescents, 61% with possible severe asthma and 58% of children with at least one exacerbation were under specialist management; only 28% of children and adolescents with uncontrolled asthma were receiving specialist care (Table 2).

For children and adolescents managed in secondary care, increased treatment intensity in terms of average daily exposed ICS dose (median dose 334 mcg (205, 468) versus 231 mcg (128, 385)) and the use of additional controllers was seen. However, 71% of children and adolescents exposed to high dose ICS were managed solely in primary care, with only small differences in annual SABA use was seen (primary care median annual doses 200 (100, 300) versus 200 (100, 420) in secondary care) (Table 3).

**Table 3:** Differences in treatment intensity and disease control in primary care and specialist management for 29,851 children aged 2–17 years with actively treated asthma from a nationwide cohort

	Primary care (N = 23,987)^a^	Specialist care (N = 5864)^a^
ICS dose		
Below low	5885 (25%)	562 (9.6%)
Low	7793 (32%)	1748 (30%)
Moderate	6778 (28%)	2127 (36%)
High	3531 (15%)	1427 (24%)
Average daily exposed dose (mcg beclomethasone)	231 (128, 385)	334 (205, 468)
Add-on therapies		
Long-acting beta_2_-agonists	5139 (21%)	1755 (30%)
Long-acting antimuscarinics	34 (0.1%)	36 (0.6%)
Leukotriene receptor antagonists	4581 (19%)	1858 (32%)
Asthma control		
Annual SABA use	200 (100, 300)	200 (100, 420)
High annual SABA use^b^	3430 (14%)	1320 (23%)
Exacerbations		
Any exacerbation(s)	999 (4.2%)	1354 (23%)
Moderate exacerbation(s)	394 (1.6%)	222 (3.8%)
Severe exacerbation(s)	610 (2.5%)	1197 (20%)
Near fatal exacerbation(s)	29 (0.1%)	35 (0.6%)
GINA 2020 exacerbation criteria	682 (2.8%)	1228 (21%)

*SABA* short-acting bronchodilator

^a^Statistics presented: n (%); median (IQR)

^b^Defined as > 400 annual doses of SABA for ages 0–11, > 600 for ages 12 and above

## Discussion

In the present study, we demonstrate a high prevalence (21.5%) of at-risk asthma among children, based on prevalence of traditional classifications of asthma severity, exacerbations, and control. The prevalence of severe paediatric asthma demonstrated an age-dependent decrease from 6.5 to 3.0% from preschool years to adolescence. In addition, we identify an exacerbation-prone group, particularly among school age children and adolescents classified as having mild-to-moderate disease, which comprised of 7.9% of the cohort. Finally, we demonstrate an unmet need in access to specialist care for severe asthma and, which is even larger among children with poor disease control and/or exacerbations among those with well-controlled mild-to-moderate asthma. In light of the limitations of using a national health registry, our approach of defining asthma disease burden in three different ways, that of severity, poor control, and of exacerbation-prone disease, allowed for a robust analysis of registry-based data.

For children aged 2–11 years, our observed prevalence of possible severe asthma ranging from 6.5–4.9%, is comparable to previous studies (2.1–10%) [18, 19]. Although methodological variations make direct comparisons difficult, the present study uses international GINA criteria and temporally averaged data in a nationwide cohort not dependent on patient-report to provide robust estimates free from seasonality-, recall- and selection bias. A notable drop in the prevalence of possible severe asthma is seen within the adolescent age-group at 3.0%, attributable to the method used for GINA Step-scoring incorporating adherence, which is known to decrease during adolescence [20]. The prevalence of adolescents experiencing exacerbations was found to be 7.1%, similar to their younger peers and slightly higher than the prevalence of possible severe asthma in young adults aged 18–45 at 5.7% [16]. Together, these details highlight high prevalence of severe asthma in Danish children, that decreases with age, in keeping with the known effect of age on childhood asthma [21].

Using annual SABA doses as a surrogate marker of poor disease control, we found that 16% of children had poor disease control, similar to the 15% reported in a study based on parent report in a Swedish cohort [22], yet markedly lower than in recent pharmacoepidemiologic cohort where 45–26% of children and adolescents redeemed 3 or more annual canisters of SABA [23]. Differences may be attributable to differences in canister sizes, follow-up duration as well as differences in symptoms perceived by parents versus administered pharmacological treatment. High SABA use has been associated with future exacerbation risk in children [23–25], and as such the prevalence of high SABA use in the present cohort warrants attention. While our findings are unavoidably confounded by inclusion of ICS-treated transient wheezing amongst the youngest children, the high SABA use even in this population is reflective of high disease burden. Even if used for viral induced episodes, given that viral infections are the primary trigger and underlying factor associated with asthma onset and persistence [26], our findings suggest that excess SABA use may be a predictor of future (severe) asthma, and is a factor to be incorporated in the decision to refer to a specialist. It is important to acknowledge that while we report annualized use, in keeping with the NHLBI and GINA guidelines, paediatric asthma is episodic so likely these doses were used in clusters of time. Lack of access to self-reported medication use or of medication use monitoring precludes our ability to address the time of use of medication. However, similar to the decline in severe asthma, an age-dependent decline in prevalence of both poor disease control and exacerbations is seen within the present cohort, which mirrors the decline of childhood wheezing with age [27] and provides estimates of poor asthma control in children and adolescents aged 6–17 years that are closer to earlier studies [22].

While uncontrolled childhood asthma leads to reduced school attendance, reduced physical fitness and quality of life [28, 29], we identified an exacerbation-prone cohort in all three age groups that had well controlled, mild to moderate asthma. Furthermore, a substantial number of children and adolescents with at-risk asthma had poorly controlled, exacerbation-prone asthma, demonstrating distinct clinical phenotypes with a high exacerbation burden. There is emerging literature on the ability of bronchodilator reversibility and exacerbations to predict poor disease control, future events and exacerbations that contribute to lung function deficits [11, 12, 30]. Furthermore, irregular check-ups have been associated with exacerbation-prone asthma [28]. These observations in the context of our cohort highlights the importance of identifying this subset of children and ensuring appropriate referral to specialist care to prevent development of these complications to provide young individuals with asthma optimal circumstances for their coming adulthood in terms of pulmonary health.

It is of note that there is substantial under-referral for specialist care, even in a population with universal health care. Children and adolescents using high-dose ICS or experiencing poor disease control are common outside of specialist care, whereas exacerbations seem to result in more frequent referrals to specialists in the present cohort. Given the patterns we observe along with the previously mentioned risks with uncontrolled childhood asthma, we speculate that better disease classification on the severity and control criteria, more regular follow up of children who have high severity with or without poor control would help the primary care physician identify those at-risk earlier and potentially refer for specialist care sooner. The effect of specialist care on long-term paediatric asthma outcomes, although investigated to a limited extent in Northern European settings, demonstrate beneficial effects [31, 32]. Additional studies are needed to further support the role of specialist care among those with high disease burden.

### Limitations

We acknowledge that there are several limitations to our observational study related to study design using a nationwide registry and availability of data. First, the inclusion criteria are based on redemption of ICS and thus excludes SABA-only and LTRA-only treated children as well as children with episodic/seasonal use of ICS due to mild asthma. Along these lines, we used the redeemed ICS doses rather than the prescribed ICS doses as the inclusion criteria and do not have information on the doses that were administered. While this limitation is inherent to the study design based on use of a nationwide registry, use of redeemed rather than prescribed doses is likely to be closer to administered doses, given that prescriptions that are not redeemed are not being administered to children. Second, the official GINA 2020 guidelines depend on prescribed dose of controller therapy to assign treatment steps, in contrast to the exposed dose used in the present study. Third, while the lower age bound used in the inclusion criteria (2 years of age) limits the inclusion of viral wheezing/asthmatic bronchitis into the cohort, some degree of ICS-treated viral wheezing cannot be excluded, though early, exacerbating viral wheezing is predictive of ICS-treated asthma in adolescents [33]. Fourth, the use of SABA redemption as a measure of disease control is limited given the lack of access to GINA self-reported symptoms and may incorrectly flag SABA use before physical activity or for viral-related symptoms as poor disease control. Finally, primary care diagnosis codes are unavailable in Danish nationwide registries, impacting tracking of asthma-related assessments in primary care. However, the prescription of ICS to children without prior physical clinical assessment is highly discouraged, and children receiving ICS treatment are expected to attend regular clinical assessments. Hence, ICS prescription without a physical assessment is unlikely to be a relevant proportion of our cohort. Despite these limitations, we would like to highlight the strengths of the REASSESS Youth cohort, which include a high degree of data completeness, universal linkage between children and parents as well as robust and non-biased data capture due to legal requirements to submit data to Statistics Denmark from the various sources used, which together make it a robust health care registry to investigate disease burden and healthcare access for paediatric asthma in universal health care system.

## Conclusion

Throughout childhood and adolescence, there was a high prevalence of severe asthma and poor control, although their prevalence declined with age. We demonstrate a large unmet need for specialist care among children with at-risk asthma, particularly among those with poorly controlled asthma, even in a system with free-to-access, tax-funded healthcare.

## Supplementary Information

**Additional file 1: Table S1.** Asthma Drug Formulations. **Table S2.** ICS Strength Calculations. **Table S3.** Differences in treatment intensity for 29,851 children aged 2-17 years with actively treated asthma, stratified by sex, from a nationwide cohort. **Table S4.** Differences in disease control and exacerbation burden for 29,851 children aged 2-17 years with actively treated asthma, stratified by sex, from a nationwide cohort.

**Additional file 2: Figure S1.** Distribution of GINA 2020 Treatment Steps in a nationwide cohort of 29,851 children aged 2–17 years with actively treated asthma, stratified by age, sex and place of asthma management.

## Data Availability

All data and supporting material are available through Statistics Denmark upon application, however approval from data sources may be required as per Danish law.

## Abbreviations

ATC: Anatomical therapeutic chemical (code)
ICS: Inhaled corticosteroids
GINA: Global initiative for asthma
SABA: Short-acting beta-2 agonist
ICD: International classification of diseases
IQR: Interquartile range
LABA: Long-acting beta-2 agonist
LTRA: Leukotriene receptor antagonists
