# Supplementary material for: Burden and unmet need for specialist care in poorly controlled and severe childhood asthma in a Danish nationwide cohort
Source: Respir Res. 2023 Jun 27;24:173. doi: 10.1186/s12931-023-02482-7 (PMC10304602; doi:10.1186/s12931-023-02482-7)
Supplement: Supplementary file 1 — Additional file 1: Table S1. Asthma Drug Formulations. Table S2. ICS Strength Calculations. Table S3. Differences in treatment intensity for 29,851 children aged 2-17 years with actively treated asthma, stratified by sex, from a nationwide cohort. Table S4. Differences in disease control and exacerbation burden for 29,851 children aged 2-17 years with actively treated asthma, stratified by sex, from a nationwide cohort. [file 12931_2023_2482_MOESM1_ESM.docx]

**ADDITIONAL MATERIALS**

**Burden and unmet need for specialist care in poorly controlled and severe childhood asthma in a Danish nationwide cohort**

Kjell Erik Julius Håkansson (MD PhD)^1*^, Silvia Cabrera Guerrero (MD)^2*^, Vibeke Backer (DMSc)^3^, Charlotte Suppli Ulrik (DMSc)^1,4^, Deepa Rastogi (MBBS)^2,5^

^1^Department of Respiratory Medicine, Copenhagen University Hospital - Hvidovre, Kettegård Allé 30, 2650, Hvidovre, Denmark.
^2^Division of Pulmonary and Sleep Medicine, Children's National Health System, 111 Michigan Ave NW, Washington, DC 20010, USA.
^3^Department of Otorhinolaryngology, Copenhagen University Hospital - Rigshospitalet, Blegdamsvej 9, 2100, Copenhagen, Denmark
^4^Institute of Clinical Medicine, University of Copenhagen, Blegdamsvej 3B, 2200, Copenhagen, Denmark.
^5^Pediatrics, Genomics and Precision Medicine, George Washington University School of Medicine and Health Sciences, 2300 I St NW,Washington, DC 20052, USA.
^*^These authors contributed equally.

**Corresponding author full contact details:**Prof. Deepa Rastogi (drastogi@childrensnational.org)
Division of Pulmonary and Sleep Medicine, Children's National Health System
111 Michigan Ave NW, Washington DC 20010, USA

**Keywords:** pediatric asthma, population cohort, exacerbations

***Table S1 - Asthma Drug Formulations***

Patients are considered to be actively treated with a drug on redemption of 2 or more prescriptions of a drug group during the study period. Drug groups as defined by ATC-categories.

| *Drug* | *ATC-codes* |
| --- | --- |
| *ICS-only* | R03BA[01-09] |
| *ICS + Long-acting Beta-agonists (LABA)* | R03AK0[6-9] or R03AK[10-14] or  The combination of ICS-only and LABA-only |
| *ICS + LABA + Long-acting antimuscarinics (LAMA)* | R03AL1[1-2] or R03AL0[8-9]  *or*  *The combination of:*  *ICS-only and LABA+LAMA-combination*  *or*  *ICS-only and LABA-only and LAMA-only* |
| *Stand-alone LABA* | R03AC1[0-9] |
| *Stand-alone LAMA* | R03BB0[4-7] |
| *Combination LAMA-LABA* | R03AL0[3-7], R03AL10 |
| *Leukotriene Receptor Antagonist (LTRA)* | R03DC |
| *Theophylline* | R03DA04 |
| *Systemic corticosteroids* | H02AB04, H02AB06 |

***Table S2 - ICS Strength***

Due to the lack of a universal dose equivalence chart in ICS treatment, a dose-equivalence estimation coefficient has been used based on the GINA 2020 Guideline dosing table using standard particle CFC-free beclomethasone (bec.) as baseline and the comparator (comp.) ICS as follows:

$$\frac{\frac{Mean comp. boundry, Low}{Mean bcm. boundry, Low}+\frac{Mean comp. boundry, Medium}{Mean bcm. boundry, Medium}+\frac{Mean comp. boundry, High}{Mean bcm. boundry, High}}{3}$$

Resulting coefficients for comparable doses are as such:

| *Step* | *Coefficient* | *ATC-codes* |
| --- | --- | --- |
| *CFC-free, standard particle beclomethasone* | *1*bec* | *R03BA01, R03AK08, R03AL08 AND varenummer 40417, 154147, 443228, 95456* |
| *CFC-free, extra-fine particle beclomethasone* | *2.36*bec* | *R03BA01, R03AK08, R03AL08 EXCEPT varenummer 40417, 154147, 443228, 95456* |
| *Budesonide* | *1.18*bec* | *R03BA02, R03AK07* |
| *Ciclesonide* | *2.95*bec* | *R03BA08* |
| *Fluticasone prop.* | *2.00*bec* | *R03BA06, R03AK11, R03AK06, R03BA05* |
| *Fluticasone furoate* | *8.00*bec* | *R03BA09, R03AK10, R03AL09* |
| *Mometasone furoate* | *1.92*bec* | *R03BA07* |

**Table S3**Differences in treatment intensity for 29,851 children aged 2-17 years with actively treated asthma, stratified by sex, from a nationwide cohort.

|  | **Ages 2-5 yrs^1^** | | | **Ages 6-11 yrs ^1^** | | | **Ages 12-17 yrs ^1^** | | |
| --- | --- | --- | --- | --- | --- | --- | --- | --- | --- |
|  | **Girls  (N = 4,034)** | **Boys (N=6,067)** | **p-value** | **Girls (N=3,533)** | **Boys**  **(N=6,362)** | **p-value** | **Girls (N=4,634)** | **Boys (N=5,217)** | **p-value** |
| **GINA 2020 Step** |  |  | 0.2 |  |  | 0.052 |  |  | **0.006** |
| *Step 1* | N/A |  |  | 351 (9.9%) | 593 (9.3%) |  | 1,395 (30%) | 1,526 (29%) |  |
| *Step 2* | 2,035 (50%) | 2,953 (49%) |  | 691 (20%) | 1,196 (19%) |  | 1,2891 (28%) | 1,600 (31%) |  |
| *Step 3* | 1,292 (32%) | 1,973 (33%) |  | 1,236 (35%) | 2,135 (34%) |  | 1,331 (29%) | 1,466 (28%) |  |
| *Step 4* | 771 (18%) | 1,141 (19%) |  | 1,255 (36%) | 2,438 (38%) |  | 515 (11%) | 527 (10%) |  |
| *Step 5* | N/A |  |  | N/A |  |  | 112 (2.4%) | 98 (1.9%) |  |
| **ICS Dose** |  |  | 0.2 |  |  | 0.10 |  |  | 0.14 |
| *Below Low* | 1,002 (25%) | 1,417 (23%) |  | 414 (12%) | 693 (11%) |  | 1,395 (30%) | 1,526 (29%) |  |
| *Low* | 1,047 (26%) | 1,554 (26%) |  | 754 (21%) | 1,314 (21%) |  | 2,247 (48%) | 2,625 (50%) |  |
| *Moderate* | 1,330 (33%) | 2,038 (34%) |  | 1,370 (39%) | 2,417 (38%) |  | 832 (18%) | 918 (18%) |  |
| *High* | 659 (16%) | 1,058 (17%) |  | 995 (28%) | 1,938 (20%) |  | 160 (3.5%) | 148 (2.8%) |  |
| *Average Daily Exposed Dose (mcg beclomethasone)* | 197 (100, 329) | 201 (115, 329) | **0.022** | 267 (157, 401) | 267 (164, 412) | **0.032** | 279 (164,468) | 281 (166, 468) | 0.9 |
| **Add-on Therapies** |  |  |  |  |  |  |  |  |  |
| *Long-acting Beta_2_-agonists* | 134 (3.3%) | 210 (3.5%) | 0.7 | 774 (21%) | 1,318 (22%) | 0.5 | 2,194 (47%) | 2,231 (43%) | **<0.001** |
| *Long-acting Antimuscarinics* | 134 (3.3%) | 210 (3.5%) | 0.9 | 1 (<0.1%) | 6 (<0.1%) | 0.4 | 34 (0.7%) | 16 (0.3%) | **0.003** |
| *Leukotriene Receptor Antagonists* | 881 (22%) | 1,367 (23%) | 0.4 | 770 (22%) | 1,396 (22%) | 0.9 | 986 (21%) | 1,039 (20%) | 0.10 |

1 Statistics presented: n (%); median (IQR);
GINA – Global Initiative for Asthma, ICS – Inhaled Corticosteroids

**Table S4**Differences in disease control and exacerbation burden for 29,851 children aged 2-17 years with actively treated asthma, stratified by sex, from a nationwide cohort.

|  | **Ages 2-5 yrs^1^** | | | **Ages 6-11 yrs ^1^** | | | **Ages 12-17 yrs ^1^** | | |
| --- | --- | --- | --- | --- | --- | --- | --- | --- | --- |
|  | **Girls  (N = 4,034)** | **Boys (N=6,067)** | **p-value** | **Girls (N=3,533)** | **Boys**  **(N=6,362)** | **p-value** | **Girls (N=4,634)** | **Boys (N=5,217)** | **p-value** |
| **Asthma Control** |  |  |  |  |  |  |  |  |  |
| *Median Annual SABA Use* | 300 (100, 400) | 300 (100, 500) | **0.025** | 200 (100, 300) | 200 (100, 300) | 0.3 | 100 (50, 260) | 100 (30, 250) | **0.011** |
| *Any Exacerbation(s) During Follow-up*^2^ | 251 (6.2%) | 506 (8.3%) | **<0.001** | 293 (8.3%) | 602 (9.5%) | 0.052 | 374 (8.1%) | 327 (6.3%) | **<0.001** |
| **Possible Severe Asthma^3^** | 169 (4.2%) | 327 (5.4%) | **0.006** | 205 (5.8%) | 437 (6.9%) | **0.039** | 154 (3.3%) | 138 (2.6%) | **0.048** |

1 Statistics presented: n (%); median (IQR)
2 Moderate exacerbations defined as redemption of oral prednisolone at 37,5mg for at least five days, severe as hospitalization with an asthma diagnosis and near-fatal as admission to an intensive care unit with an asthma diagnosis. Exacerbation-prone asthma was defined as two moderate or one severe exacerbation(s) during the follow-up period.
3 Defined as exacerbation-prone asthma with GINA Step 3+4 treatment for ages 0-11, exacerbation-prone GINA Step 4 or GINA Step 5 regardless of exacerbations for ages 12 and above.
SABA – Short-acting bronchodilator
