# Supplementary material for: Burden and unmet need for specialist care in poorly controlled and severe childhood asthma in a Danish nationwide cohort
Source: Respir Res. 2023 Jun 27;24:173. doi: 10.1186/s12931-023-02482-7 (PMC10304602; doi:10.1186/s12931-023-02482-7)
Supplement: Supplementary file 2 — Additional file 2: Figure S1. Distribution of GINA 2020 Treatment Steps in a nationwide cohort of 29,851 children aged 2–17 years with actively treated asthma, stratified by age, sex and place of asthma management. [file 12931_2023_2482_MOESM2_ESM.pdf]

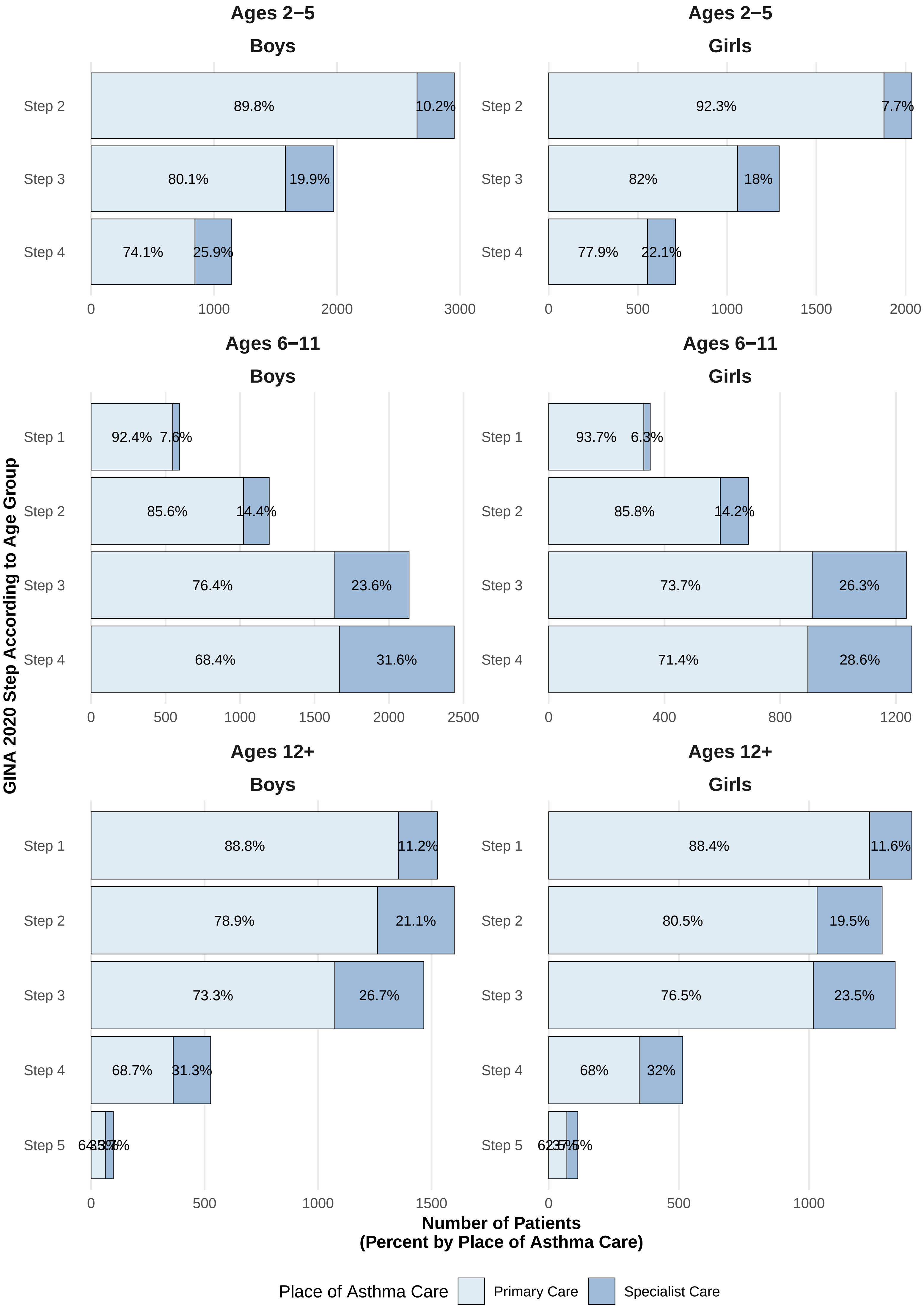

Ages 2−5
Boys
Step 2
Step 3
Step 4
89.8%
10.2%
80.1%
19.9%
74.1%
25.9%
0
1000
2000
3000
Ages 2−5
Girls
Step 2
Step 3
Step 4
92.3%
7.7%
82%
18%
77.9%
22.1%
0
500
1000
1500
2000
Ages 6−11
Boys
Step 1
Step 2
Step 3
Step 4
92.4%
7.6%
85.6%
14.4%
76.4%
23.6%
68.4%
31.6%
0
500
1000
1500
2000
2500
Ages 6−11
Girls
Step 1
Step 2
Step 3
Step 4
93.7%
6.3%
85.8%
14.2%
73.7%
26.3%
71.4%
28.6%
0
400
800
1200
Ages 12+
Boys
Step 1
Step 2
Step 3
Step 4
Step 5
88.8%
11.2%
78.9%
21.1%
73.3%
26.7%
68.7%
31.3%
0
500
1000
1500
Ages 12+
Girls
Step 1
Step 2
Step 3
Step 4
Step 5
88.4%
11.6%
80.5%
19.5%
76.5%
23.5%
68%
32%
0
500
1000
GINA 2020 Step According to Age Group
Number of Patients
(Percent by Place of Asthma Care)
Place of Asthma Care
Primary Care
Specialist Care
